# Supplementary material for: Evidence of ubiquitous Alfvén pulses transporting energy from the photosphere to the upper chromosphere
Source: Nat Commun. 2019 Aug 5;10:3504. doi: 10.1038/s41467-019-11495-0 (PMC6683129; doi:10.1038/s41467-019-11495-0)
Supplement: Supplementary file 1 — Supplementary Information [file 41467_2019_11495_MOESM1_ESM.pdf]

Supplementary Information: Evidence of Ubiquitous Alfvén Pulses Transporting Energy from the Photosphere to the Upper Chromosphere

Jiajia Liu[1,*], Chris J Nelson[2,1], Ben Snow[1], Yuming Wang[3,4], Robert Erdélyi[1,5]

[1] *Solar Physics and Space Plasma Research Centre (SP2RC), School of Mathematics and Statistics, The University of Sheffield, Sheffield, S3 7RH, UK*
[2] *Astrophysics Research Centre (ARC), School of Mathematics and Physics, Queen's University, Belfast, BT7 1NN, Northern Ireland, UK*
[3] *CAS Key Laboratory of Geospace Environment, Department of Geophysics and Planetary Sciences, University of Science and Technology of China, Hefei, Anhui 230026, China*
[4] *CAS Center for Excellence in Comparative Planetology, Hefei, Anhui 230026, China*
[5] *Department of Astronomy, Eötvös Loránd University, Budapest, Pázmány P. sétány 1/A, H-1117, Hungary*
[*] *Corresponding Author, email: jj.liu@sheffield.ac.uk*

Supplementary Note 1: SST Data

The ground-based data analysed here were targeted at a quiet region close to the disk centre, sampled by the SST/CRISP instrument between 08:07:22 UT and 09:05:44 UT on the 21st June 2012. The SST/CRISP sequence that ran during this time consisted of a single-point full-Stokes measurement at the core of the Fe I 6302.5 Å line, an eleven-point Hα line scan sampling evenly between $\pm$1.3 Å from the line core, and a nineteen-point Ca II 8542 Å line scan sampling evenly between $\pm$0.5 Å from the line core. The FOV was approximately $55'' \times 55''$, with an initial central co-ordinates of $x_{\rm c} = -3''$, $y_{\rm c} = 70''$. The data were reduced employing the Multi-Object Multi-Frame Blind Deconvolution (MOMFBD)[1] method. The standard CRISPRED pipeline[2], including additional steps to account for differential stretching[3], was also used. After applying the above reductions, the data has then a cadence of approximately 8.25 s and a pixel size of $0.059''$ ($\sim$43.6 km, resulting in a FOV of $\sim$40.6 Mm$\times$40.6 Mm).

Supplementary Note 2: Governing Equations of the Numerical Simulation and the Construction of the Single Magnetic Flux Tube

The numerical simulation used in this work has been performed employing SAC[4], which solves the full ideal, compressible MHD equations for a perturbation within a gravitationally stratified background atmosphere. The governing equations are given by:

$$
\begin{aligned}
&\frac{\partial \rho}{\partial t} + \nabla \cdot [\mathbf{v}(\rho_{\mathbf{b}} + \rho)] = 0 + D_\rho(\rho), \\
&\frac{\partial [(\rho_b + \rho)\mathbf{v}]}{\partial t} + \nabla \cdot [\mathbf{v}(\rho_b + \rho)\mathbf{v} - \mathbf{B}\mathbf{B}] - \nabla [\mathbf{B}\mathbf{B_b} + \mathbf{B_b}\mathbf{B}] + \nabla p_t \\
&\qquad = \rho \mathbf{g} + \mathbf{D}_{\rho v} [(\rho_b + \rho)\mathbf{v}], \\
&\frac{\partial e}{\partial t} + \nabla \cdot [\mathbf{v}(e + e_b) - \mathbf{B}\mathbf{B} \cdot \mathbf{v} + \mathbf{v} p_t] - \nabla [(\mathbf{B}\mathbf{B_b} + \mathbf{B_b}\mathbf{B}) \cdot \mathbf{v}] + p_{tb} \nabla \mathbf{v} - \mathbf{B_b}\mathbf{B_b} \nabla \mathbf{v} \\
&\qquad = \rho \mathbf{g} \cdot \mathbf{v} + D_e(e), \\
&\frac{\partial \mathbf{B}}{\partial t} + \nabla \cdot [\mathbf{v}(\mathbf{B} + \mathbf{B_b}) - (\mathbf{B} + \mathbf{B_b})\mathbf{v}] = 0 + \mathbf{D_B}(\mathbf{B}), \\
&p_t = p_k + \mathbf{B}^2/2 + \mathbf{B_b}\mathbf{B}, \\
&p_k = (\gamma - 1)\left[e - (\rho_b + \rho)\mathbf{v}/2 - \mathbf{B_b}\mathbf{B} - \mathbf{B}^2/2\right], \\
&p_{tb} = p_{kb} + \mathbf{B_b}^2/2, \\
&p_{kb} = (\gamma - 1)\left(e_b - \mathbf{B_b}^2/2\right).
\end{aligned} \tag{1}
$$

Here, ρ, $\mathbf{v}$, e, $\mathbf{B}$, p_k and p_t are the perturbed density, perturbed velocity vector, perturbed energy density per unit volume, perturbed magnetic field vector, perturbed kinetic pressure and perturbed total pressure, respectively. γ is the gas adiabatic index, set to be $5/3$ in the simulation. g is the gravitational field vector, set to be -274 m s^{-2} along the z-direction. Subscript b denotes background parameters. Sub-grid numerical diffusion and resistivity are applied to the equations as the terms D. Details of these hyperdiffusive and hyperresistive terms could be found in Equation (22) to Equation (32) in the original SAC paper[4].

The self-similar expanding open magnetic flux tube embedded into the background atmosphere, similar to flux tubes constructed and studied in previous work[5,6], has been constructed analytically from the following equations:

$$
\begin{aligned}
B_x &= -xB_{0z}(z)\frac{d\alpha}{dz}\cdot G(f),\\
B_y &= -yB_{0z}(z)\frac{d\alpha}{dz}\cdot G(f),\\
B_z &= \alpha B_{0z}(z)\cdot G(f) + B_{bz},\\
f &= \alpha\frac{r}{r_0},\\
G(f) &= e^{-\frac{1}{2}f^2}.
\end{aligned}
\tag{2}
$$

Here, B_x, B_y and B_z are the $x-$, $y-$ and $z-$component of the magnetic field of the flux tube. B_{bz} = 17.5 G is the z-component of the background magnetic field outside the flux tube. r is the distance to the centre of the flux tube. r_0 is the radial scaling and set as 39.38 km. The term $G(f)$ is set to be a Gaussian function of f in order to ensure the shape of the magnetic flux tube is consistent while it expands to balance the external pressure with the increasing height. $B_{0z}(z)$ is a function of z:

$$
\begin{aligned}
B_{0z}(z) &= \alpha B_{0z}(0),\\
\alpha &= e^{-\frac{z}{z_3}}
\end{aligned}
\tag{3}
$$

where, $B_{0z}(0)$ is the magnetic field strength of the flux tube at its bottom (800 G), and z_3 is the chomospheric scale height (0.45 Mm). The corresponding pressure and density deviations from the non-magnetic equilibrium of the background atmosphere are then calculated based on the total pressure balance[6,7]. More details could be found in Appendix B of the reference[6].

Supplementary Discussion 1: Density Variations Resulted from Alfvén Pulses

It is worth highlighting that the number of detected SOT/SST photospheric intensity swirls are around half of the number of SOT/SST chromospheric swirls. There could be multiple causes for this, including but not limited to: 1) photospheric intensity is an integration over different heights in the photosphere, meaning more noise; 2) rotating speed of photospheric intensity swirls is rather small (half of that of chromospheric swirls), as we can see from the results, meaning that many of them would not be resolved by the combination of the FLCT (which already usually underestimate the photospheric velocity by a factor of as much as three[8]) and swirl detection algorithm (which highly relies on the rotational speed); 3) there is different density inhomogeneity in the photosphere and chromosphere, meaning some swirls would not be detected if the local plasma is

not inhomogeneous enough. Exact reasons need to be confirmed using simulation data, however, this is beyond the present scope of this article.

We have found, that, ubiquitous photospheric swirls could excite Alfvén pulses which propagate upward into the upper chromosphere and result in the ubiquitously observed chromospheric swirls. Some readers might think the above scenario hard to understand because pure Alfvén waves are incompressible (meaning that they cannot result in local density concentration or rarefaction). However, even though Alfvén pulses cannot cause density perturbations, we demonstrate that they can still result in the observed density variation under the frozen-in condition when density inhomogeneity is present, employing the following toy model.

In this model, a uniform flux tube along the z-direction is constructed with the initial vertical and azimuthal magnetic field $B_z = 100$ and $B_a = 0$, respectively. An Alfvén pulse is introduced at the bottom of the flux tube at $t = 0$ and propagates upward with a constant speed (v). This Alfvén pulse introduces an azimuthal magnetic field perturbation defined as:

$$\begin{aligned} B_a(z, r, t) = A \cdot \frac{r}{r_0} \cdot B \cdot \cos\left(\frac{z - z_0(t)}{d_0}\pi\right), \\ z_0(t) = v \cdot t + 0.5d_0, \\ z_0(t) - 0.5d_0 \leq z \leq z_0(t) + 0.5d_0. \end{aligned} \quad (4)$$

Here, z and t are the vertical position along the flux tube and time, respectively. $0 < A < 1$ is the amplitude ratio. r and $r_0 = 300$ are the distance to the axis of the flux tube and the radius of the flux tube, respectively. B is the total magnetic field strength (100). $d_0 = 200$ is the vertical extension of the magnetic field perturbation. The plasma density (ρ) inside the flux tube is inhomogeneous, and is defined by:

$$\rho(\vartheta) = 40(1 + \cos(2\vartheta)). \quad (5)$$

Here, again, r is the distance to the axis of the flux tube. ϑ is the azimuthal angle. All the above values are set for the best appearance of the visualization.

A visualization of the constructed flux tube is shown in Movie M3. Due to the frozen-in condition, the density elements rotate accordingly in the opposite direction from the twist when the Alfvén pulse passes by and therefore could be observed as a swirl in real observations. This density variation is caused by the condition that infinitesimal plasma elements are sitting fixed on given field lines under the frozen-in condition. The variation should not be mixed up by material in/out-flows that may happen, would the passing pulse not be Alfvén.

We shall notice that the above scenario is based on two basic conditions: plasma is frozen-in and there is a local density inhomogeneity. The first condition is fulfilled in the upper chromosphere, while the second is not always fulfilled for a given instrumental resolution. We demonstrate that, if a swirl is observed in the photosphere while the corresponding plasma density in the upper chromosphere is not significantly inhomogeneous, the Alfvén pulse could still be excited but no chromospheric swirl will be observed. This adds another effect into what we suggested in the main article, that, the number of photospheric swirls which were found to have their correspondences in the chromosphere should have been under-estimated.

Supplementary Discussion 2: Energy Flux of Alfvén pulses

Under the small-amplitude, short-wavelength assumption, the energy flux carried into the upper chromosphere by a single Alfvén pulse could be expressed as:

$$F_A = \rho \hat{v}^2 c_A, \tag{6}$$

where, $\rho \approx 4 \times 10^{-8}$ kg m^{-3} is the mass density at the bottom of the upper chromosphere in the simulation ($z = 1000$ km), $\hat{v} \approx$ 2 - 4 km s^{-1} is the observed average rotating speed of swirls, and $c_A \approx$ 12 km s^{-1} is the Alfvén speed at $z = 1000$ km. These measurements result in an energy flux from 1.9 to 7.7 kW m^{-2} - large enough to balance local upper chromospheric energy loss ($\sim 10^2$ W m^{-2}) in quiet regions[9]. To evaluate their contribution to the global heating, we calculate the average energy flux ($\overline{F_A}$):

$$\overline{F_A} = \frac{F_A \overline{N} \pi \overline{R}^2}{S_{FOV}}, \tag{7}$$

where, $F_A =$ 1.9 - 7.7 kW m^{-2} is the energy flux carried by a single Alfvén pulse estimated above. $\overline{N} =$ 48.2 and $\overline{R} = 0.3$ Mm are the average number of swirls in each frame and swirl effective radius in the SOT chromospheric observations. $S_{FOV} = 800$ Mm^2 is the area of the FOV of the SOT observations. $\overline{F_A}$ is, then, found to be around 33 - 131 W m^{-2}, comparable to the energy flux needed to compensate the observed excess in radiation. Moreover, there are several facts that we should also bear in mind:

First, we have found, via applying ASDA to a series of realistic MHD simulations, that, by slightly decreasing the pixel size from 39.2 km to 31.2 km, ASDA found 70% more photospheric swirls[10]. This means that the average number of swirls detected in each frame is largely under-estimated with the current resolution. The estimated total number of chromospheric swirls and

average energy flux could be extrapolated to 6.3×10^5 and 56 - 222 W m^{-2}, respectively, if the pixel resolution increases to 31.2 km.

Second, we have set a very strict criteria: any swirl candidate with expanding/shrinking speed larger than half of its rotating speed was not considered as a swirl. Many candidates which might be potential swirls have been removed due to the above criteria. For example, if we slightly loosen the criteria to keep candidates with expanding/shrinking speed less than their rotating speeds, the total number of swirls would be 4 times larger, as well as the average associated energy flux (132 - 524 W m^{-2}).

Third, We have demonstrated that, even though ASDA could keep high accuracy in determining swirl properties, many swirls would be missed when noise is present[10].

And Fourth, as per the previous discussions, the successful observation of chromospheric swirls rely on local density inhomogeneity. Some Alfvén pulses could have been missed in the observations due to insignificant density inhomogeneity.

Having considered the above effects, we demonstrate that the average energy flux contributed by Alfvén pulses estimated above (33 - 131 W m^{-2}) should be the lower limit. We shall also note that, possible reflection and dissipation may also affect the local energy budget. However, we do not see any evidence of reflection or dissipation of these Alfvén pulses in the data. This may be an interesting future direction to investigate, but is beyond the scope of the current study. Moreover, the estimation of the energy flux of the observed Alfvén pulses is based on either empirical/theoretical (mass density and Alfvén speed) or observational (the average rotating speed of swirls) results at the bottom of the upper chromosphere, thus possible reflection and dissipation during their propagation from the photosphere to the bottom of the upper chromosphere are irrelevant in the above energy flux estimation.

To conclude, Alfvén pulses introduced by the observed atmospheric intensity swirls are able to carry considerable energy into the upper chromosphere. The associated energy flux is more than enough to balance the local upper chromospheric energy losses in quiet Sun regions, while their global contribution needs to be further studied using observations with higher spatial and temporal resolutions.

Supplementary Discussion 3: Possible Relationship with Small-scale Magnetic Flux Tubes

In the numerical simulation, we have studied the propagation of an Alfvén pulse in an expanding magnetic flux tube. Thus, if we could find some (significant) correlation between photospheric swirls and small-scale vertical magnetic flux tubes, we will have more evidence about the excitation of the Alfvén pulses. However, what are the observational signatures of magnetic flux tubes? We

could be almost sure that, magnetic bright points[11] (MBPs) represent small-scale magnetic flux tubes with strong (up to kG) magnetic field. But, are there small-scale magnetic flux tubes if there is no MBP? We are afraid that the answer is yes.

Even if ignoring the above dilemma, we have found it is impossible to study the correlation between the detected swirls and MBPs using the currently available data because:

First, SST observations could provide observations of MBPs with barely enough resolution. However, unfortunately, we do not have enough Stokes observations to derive magnetic field information in our current available dataset, even if we ignore the influence of the seeing effect on the observation of MBPs.

And second, magnetic field data observed by the Helioseismic and Magnetic Imager[12] (HMI) onboard the Solar Dynamics Observatory (SDO) might be a candidate, however, its resolution (with a pixel size of more than 440 km, $\sim$10 times of that of the utilized SST observations) seems too large. Given that the typical radius of a photospheric MBP is around 100 km[13], its magnetic field would be averaged to as low as 30 to 60 G in HMI observations, if its original magnetic field strength is a typical value of 500 to 1000 G. It means, if we see a bright pixel with magnetic field just above 3 times of the observational error (10 G)[14], we may have no idea whether it is an averaged MBP, or a weak region with no MBP, or just simply within the 3σ error.

Ignoring the above effects for a moment, we have done a series of tests using HMI line-of-sight (LOS) magnetograms. Supplementary Figure 7 shows an example of the absolute LOS magnetic field with a scale from 0 (white) to 50 G (black), in the FOV of the corresponding SST observations. HMI data has been aligned to match the location and orientation of SST observations, using information derived from feature comparison between SST Fe I 6302 Å wideband and AIA 1700 Å observations. We can see how coarse the HMI observation is and how small-scale magnetic elements are smoothed over pixels.

Red and blue contours are the detected SST photospheric intensity swirls. Now, we use an extremely loose criterion: any swirl with even one point having absolute magnetic field strength above 30 G is considered to have a strong magnetic field (and thus marked as corresponding to a magnetic flux tube), and is contoured out with solid lines. All others are contoured out with dashed lines. As we can see, there is only 1 swirl corresponding to strong magnetic field in the HMI image.

We have further studied all 77 frames of the HMI observations during the period of the utilized SST observations in this work, by exploring their correlation with photospheric intensity swirls detected in their closest (in time) SST frames. It turns out that, only 3.3% of the swirls have been found to correspond to strong HMI magnetic field regions. As a comparison, we further did a Monte-Carlo test by comparing each HMI observation with a random (in time) SST swirl detection

result. Unsurprisingly, again, 3.3% swirls have been found to correspond to strong HMI magnetic field regions. All the above results indicate that, HMI observations are too coarse to be used.

To conclude, we are not able to see any reliable solution for this particular problem using solar observations at the current stage. As far as we are aware, there are possibly two ways to study this particular problem: 1) applying swirl detection and MBP detection algorithms to realistic simulation data (for example Bifrost). This will be one of our future avenues of work, however, it would require access to Bifrost simulation data and the development of (or use of if there is openly available) an automated MBP detection algorithm; 2) using the high-resolution magnetic field observations from the Daniel K. Inouye Solar Telescope (DKIST) would be another good choice.

Supplementary Figures

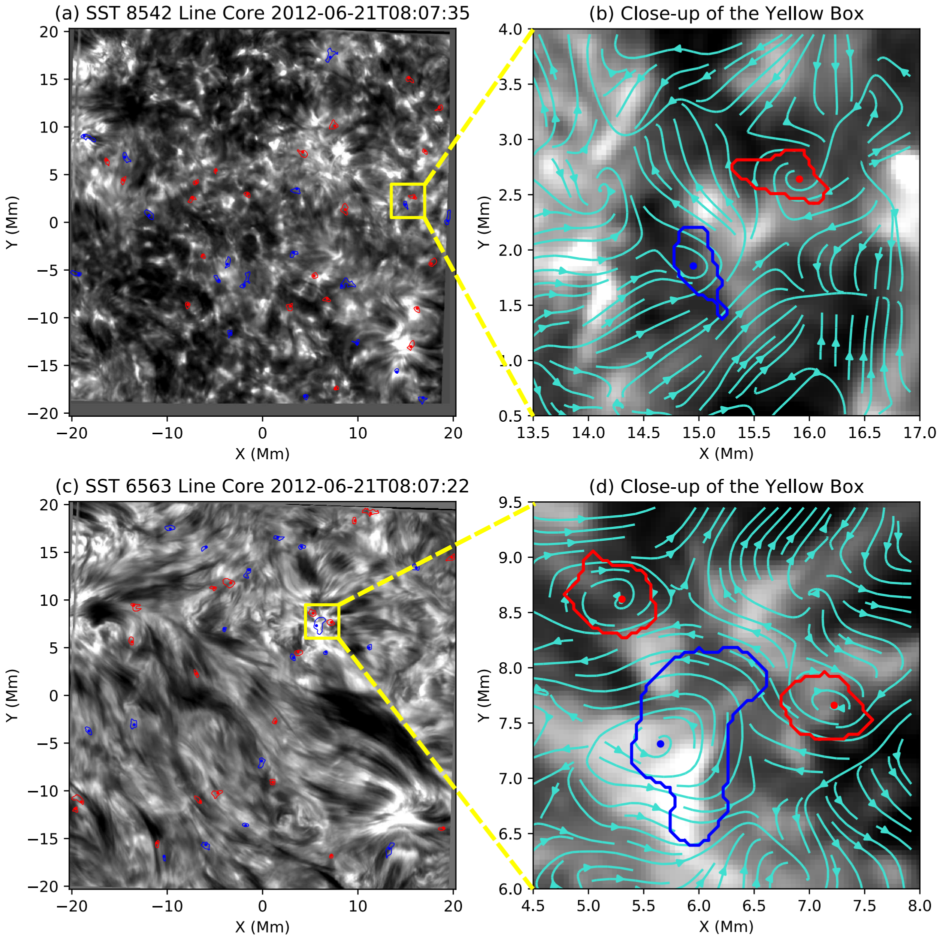

Supplementary Figure 1: **Chromospheric swirls detected from the SST observations.** Panel **(a)** and **(b)** are observations at the Ca II 8542 Å line core, and panel **(c)** and **(d)** are observations at the Hα 6563 Å line core. The intensity observations are shown as the white-black background in all panels. Swirls, with positive (negative) rotating direction are denoted in blue (red). Contours and dots are their edges and centres, respectively. Turquoise arrows in panel **(b)** and **(d)** represent tracked velocity field by the FLCT method. Source data are provided in the Source Data file.

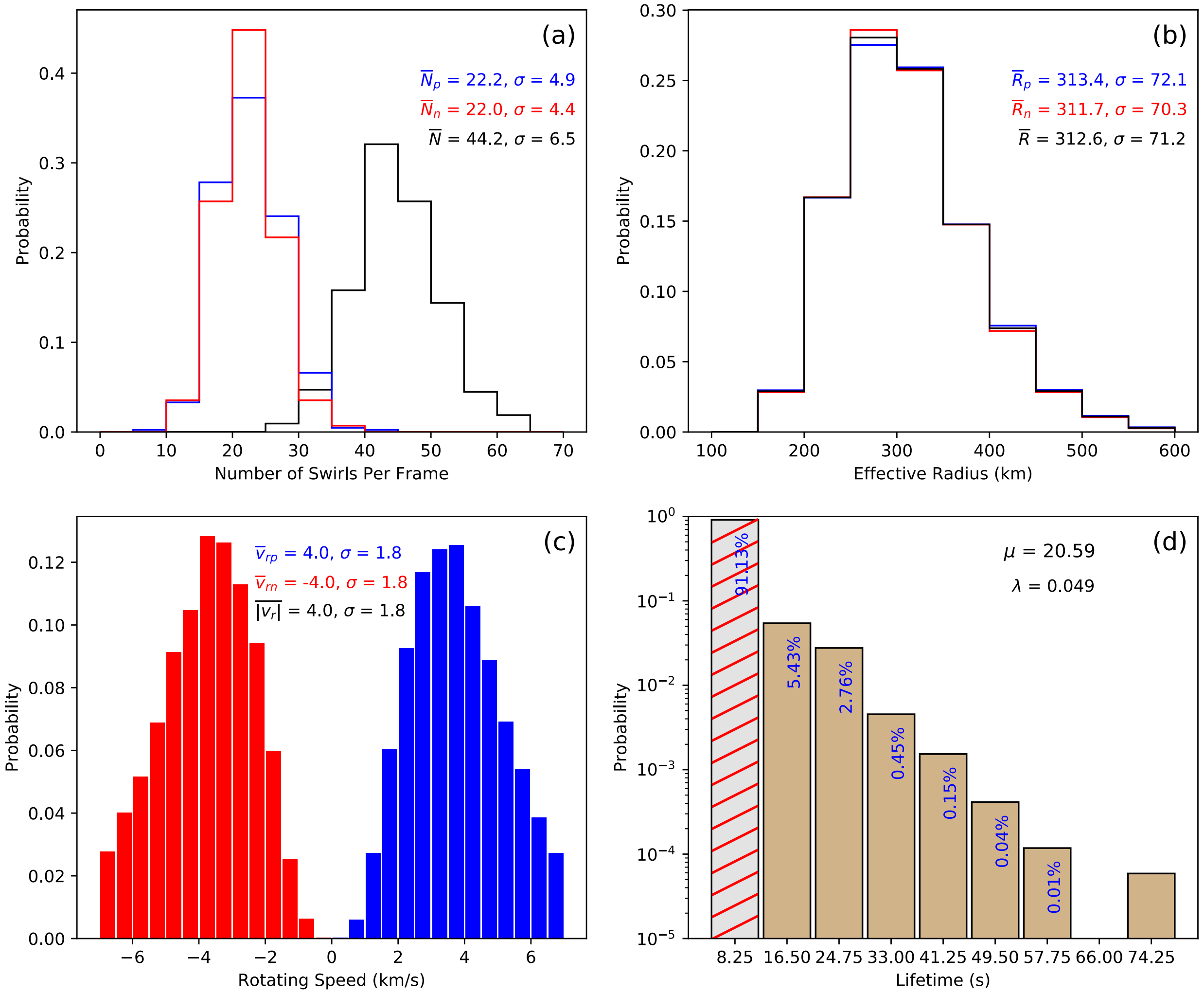

Supplementary Figure 2: **Statistics of swirls detected from the SST Ca II line core observations.** Panel **(a)** denotes the distribution of number of swirls per frame, with **(b)** the effective radii, **(c)** the average rotating speeds at edges, and **(d)** the lifetimes of all swirls. Blue (red) curves, bars and texts in the first three panels represent results of positive (negative) swirls. Black curves and texts are the results of all swirls. μ and λ in panel **(d)** are the expectation and maximum likelihood estimation of the exponential rate parameter of the lifetime, respectively. The left most bar is stripped, because lifetimes less than twice of the cadence are not measured but estimated given the limitation on the cadence. It has also been excluded when estimating μ and λ. Source data are provided in the Source Data file.

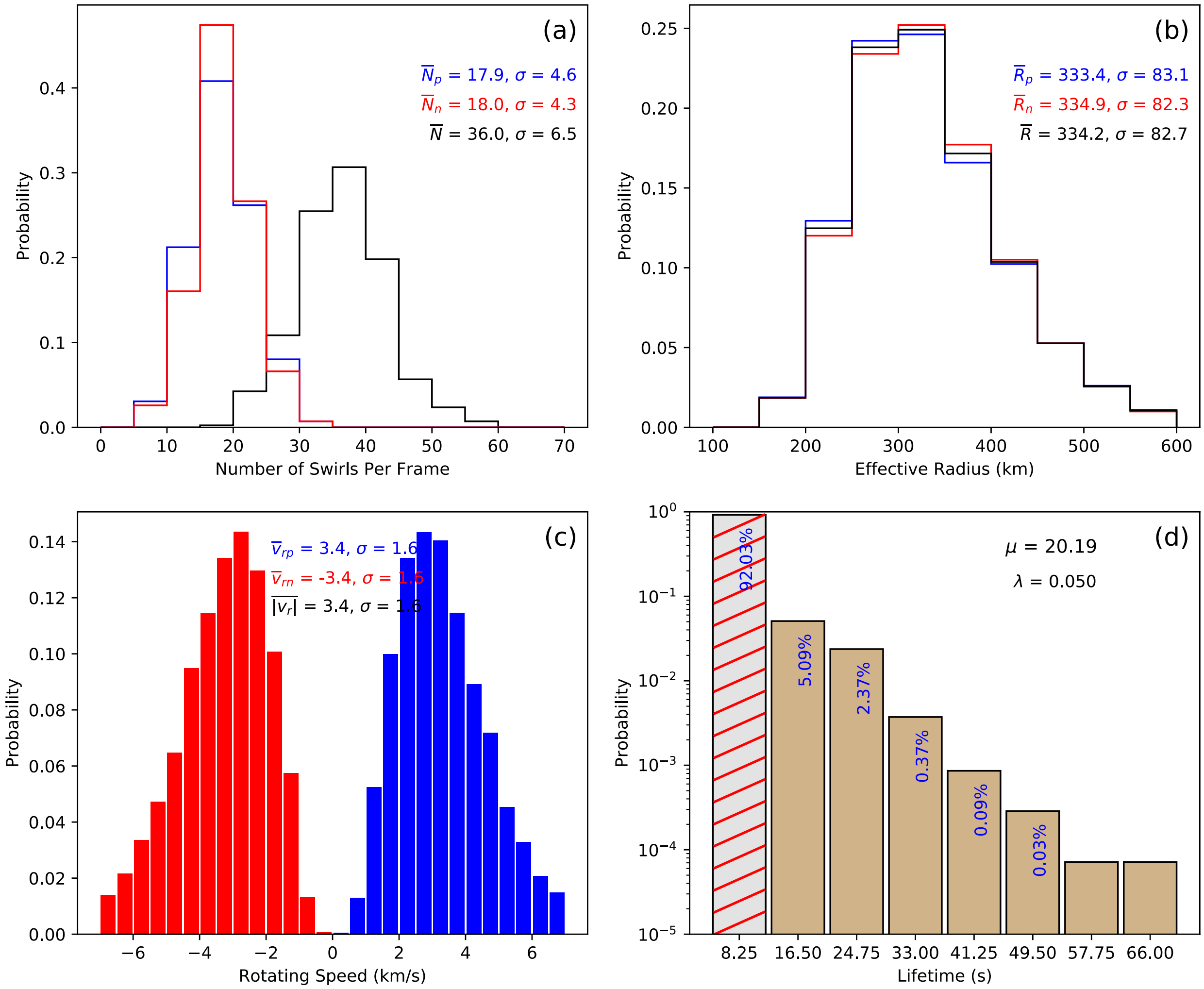

Supplementary Figure 3: **Statistics of swirls detected from the SST Hα line core observations.** See the legend of Supplementary Figure 2 for explanations of symbols, bars and colors. Source data are provided in the Source Data file.

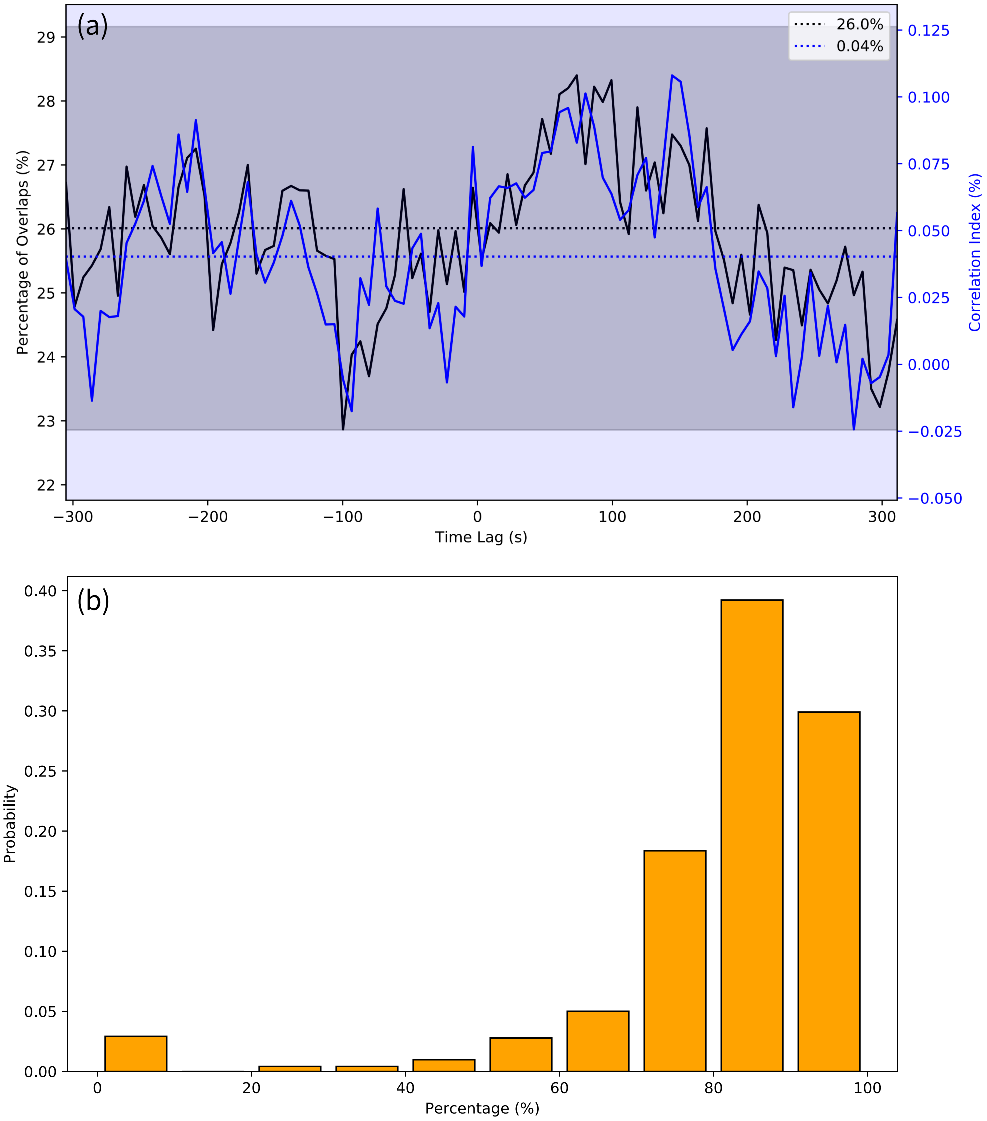

Supplementary Figure 4: **Correlation between SOT photospheric and chromospheric swirls**. Panel **(a):** Same as Fig. 3(b) but on a pre-shuffled dataset. We randomly shuffled the SOT Ca II H chromospheric observations once, before performing the calculation of the CI and overlap for varying time lags. No significant peaks could be found above the 3σ levels. Panel **(b):** Histogram of percentage of swirls (in each SOT photospheric frame) which could be found to correspond to original (not pre-shuffled) SOT Ca II H chromospheric swirls within a time lag range of 100 s to 160 s. Source data are provided in the Source Data file.

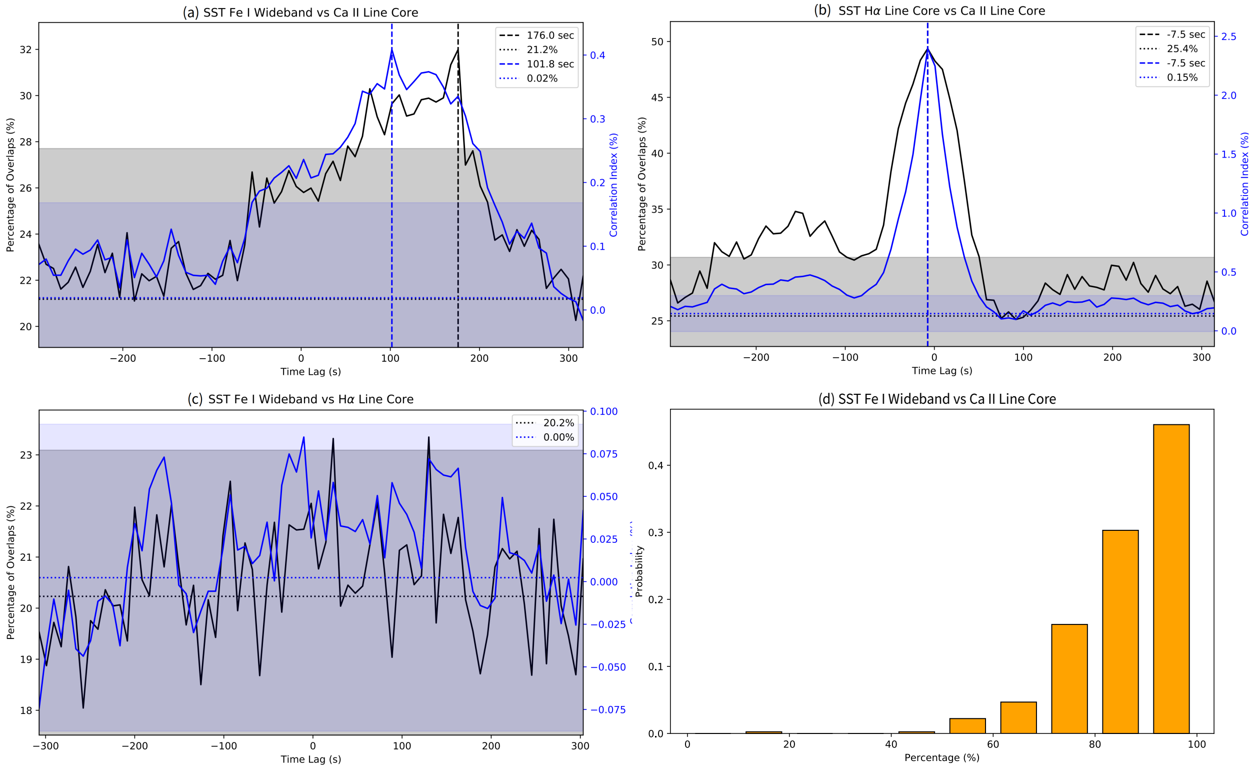

Supplementary Figure 5: **Correlations between swirls detected from different SST lines.** Panel **(a)**: Fe I wideband photospheric observations vs. Ca II 8542 Å line core observations. Panel **(b)**: Hα 6563 Å line core observations vs. Ca II 8542 Å line core observations. Panel **(c)**: Fe I wideband photospheric observations vs. Hα 6563 Å line core observations. Meanings of colours and annotations are similar to those in Fig. 3(b) in the main article. Shadows in panels **(a)** and **(b)** are the 5σ ranges, while shadows in panel **(c)** are the 3σ ranges. Panel **(d):** Histogram of percentage of swirls (in each SST Fe I wideband photospheric frame) which could be found to correspond to SST Ca II line core chromospheric swirls within a time lag range of 100 s to 160 s. Source data are provided in the Source Data file.

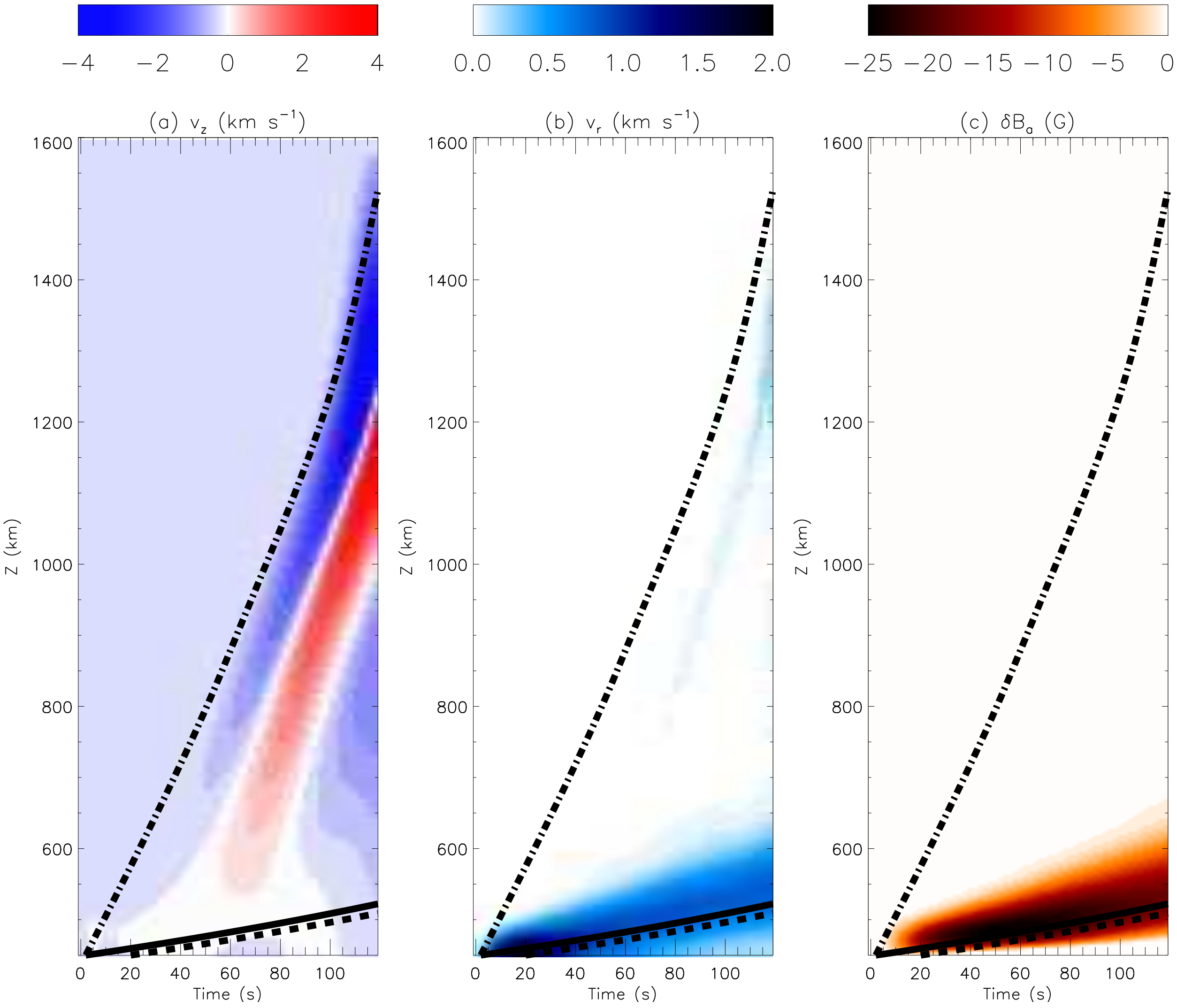

Supplementary Figure 6: **Time-distance plots of a slit located outside the flux tube.** The slit is located at a coordinate of around $x = 395$ km, $y = 395$ km along the z direction. See Fig. 4 for explanations of symbols, colors and curves. Source data are provided in the Source Data file.

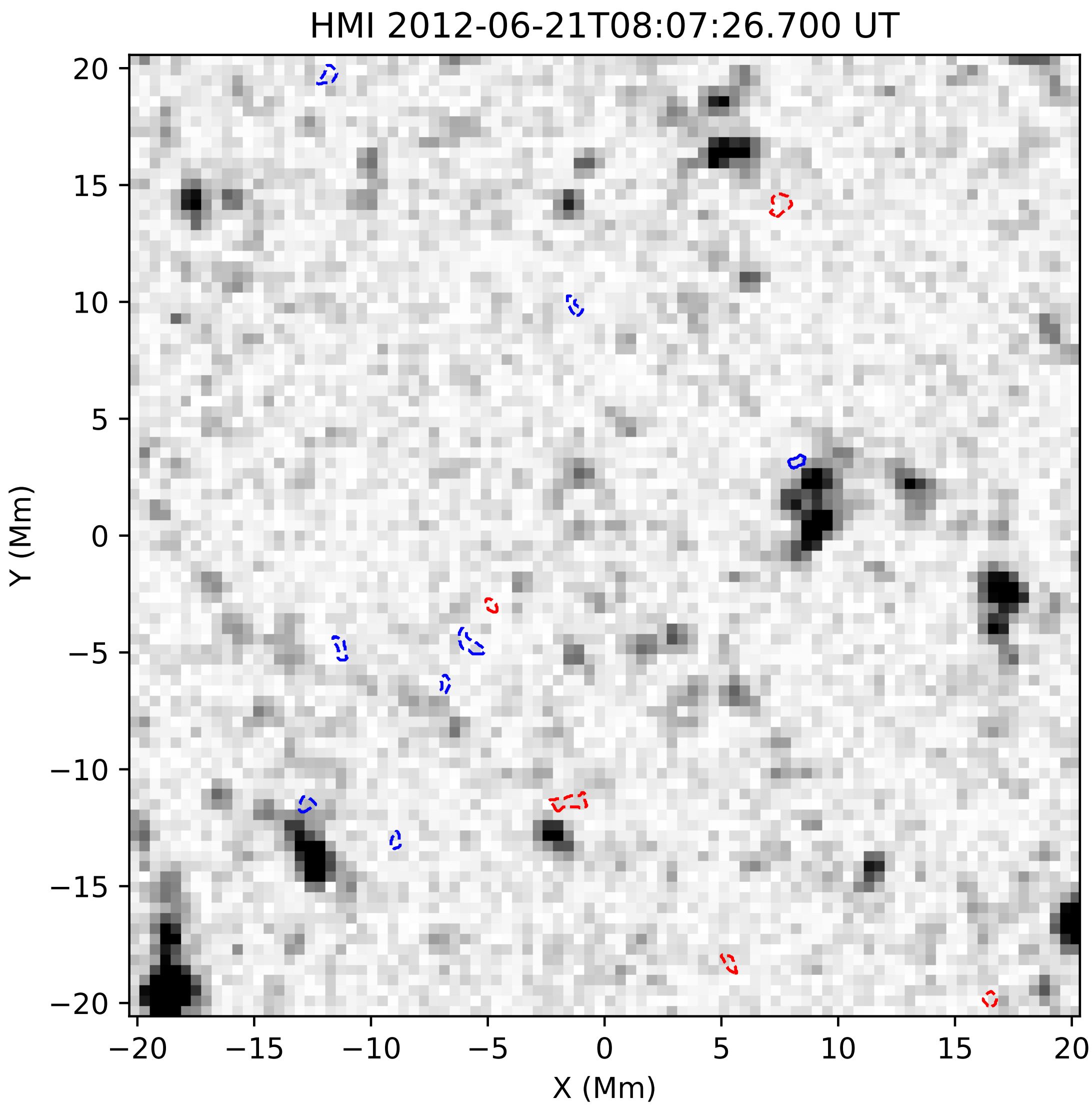

Supplementary Figure 7: **HMI LOS magnetogram and SST photospheric swirls**. Background: HMI LOS magnetogram at 08:07:26 UT in the FOV of the studied SST observations in the paper, with a scale of the absolute LOS magnetic field strength from 0 G (white) to 50 G (black). Red and blue contours: clockwise and counter-clockwise photospheric intensity swirls detected from SST Fe I 6302 Å wideband observations at almost the same time. Any swirl with even one point having absolute magnetic field strength above 30 G is contoured out with solid lines. All others are contoured out with dashed lines. Source data are provided in the Source Data file.

Supplementary References

1. van Noort, M., Rouppe van der Voort, L. & Löfdahl, M. G. Solar Image Restoration By Use Of Multi-frame Blind De-convolution With Multiple Objects And Phase Diversity. *Solar Phys.* **228**, 191–215 (2005).

2. de la Cruz Rodríguez, J., Löfdahl, M. G., Sütterlin, P., Hillberg, T. & Rouppe van der Voort, L. CRISPRED: A data pipeline for the CRISP imaging spectropolarimeter. *A&A* **573**, A40 (2015).

3. Henriques, V. M. J. Three-dimensional temperature mapping of solar photospheric fine structure using Ca II H filtergrams. *A&A* **548**, A114 (2012).

4. Shelyag, S., Fedun, V. & Erdélyi, R. Magnetohydrodynamic code for gravitationally-stratified media. *A&A* **486**, 655–662 (2008).

5. Fedun, V., Shelyag, S., Verth, G., Mathioudakis, M. & Erdélyi, R. MHD waves generated by high-frequency photospheric vortex motions. *Ann. Geophys.* **29**, 1029–1035 (2011).

6. Gent, F. A., Fedun, V., Mumford, S. J. & Erdélyi, R. Magnetohydrostatic equilibrium - I. Three-dimensional open magnetic flux tube in the stratified solar atmosphere. *MNRAS* **435**, 689–697 (2013).

7. Mumford, S. J. & Erdélyi, R. Photospheric logarithmic velocity spirals as MHD wave generation mechanisms. MNRAS **449**, 1679–1685 (2015).

8. Verma, M., Steffen, M. & Denker, C. Evaluating local correlation tracking using CO5BOLD simulations of solar granulation. *A&A* **555**, A136 (2013).

9. Withbroe, G. L. & Noyes, R. W. Mass and energy flow in the solar chromosphere and corona. *Ann. Rev. A&A* **15**, 363–387 (1977).

10. Liu, J., Nelson, C. J. & Erdélyi, R. Automated Swirl Detection Algorithm (ASDA) and Its Application to Simulation and Observational Data. *Astrophys. J.* **872**, 22–35 (2019).

11. Almeida, S. J. & Bonet, J. A. & Viticchi, B. & Del Moro, D. Magnetic Bright Points in the Quiet Sun. *Astrophys. J.* **715**, 26–29 (2010).

12. Scherrer, P. H., Schou, J., Bush, R. I., Kosovichev, A. G., Bogart, R. S., Hoeksema, J. T., Liu, Y., Jr. Duvall T. L., Zhao, J., Title, A. M., Schrijver, C. J., Tarbell, T. D. & Tomczyk, S. The Helioseismic and Magnetic Imager (HMI) Investigation for the Solar Dynamics Observatory (SDO). *Solar Phys.* **275**, 207–227 (2012).

13. Keys, P. H., Mathioudakis, M., Jess, D. B., Shelyag, S., Christian, D. J. & Keenan, F. P. Tracking magnetic bright point motions through the solar atmosphere. *MNRAS* **428**, 3220–3226 (2013).

14. Liu, Y., Hoeksema, J. T., Scherrer, P. H., Schou, J., Couvidat, S., Bush, R. I., Duvall, T. L., Hayashi, K., Sun, X. & Zhao, X. Comparison of Line-of-Sight Magnetograms Taken by the Solar Dynamics Observatory/Helioseismic and Magnetic Imager and Solar and Heliospheric Observatory/Michelson Doppler Imager. *Solar Phys.* **279**, 295–316 (2012).
