# Supplementary material for: Evidence of ubiquitous Alfvén pulses transporting energy from the photosphere to the upper chromosphere
Source: Nat Commun. 2019 Aug 5;10:3504. doi: 10.1038/s41467-019-11495-0 (PMC6683129; doi:10.1038/s41467-019-11495-0)
Supplement: Supplementary file 3 — Description of Additional Supplementary Files [file 41467_2019_11495_MOESM3_ESM.docx]

**Description of Supplementary Files**

**File Name:** **Supplementary Movie 1**

**Description:** Visualization of the numerical simulation. A snapshot at t=93 s is shown in Fig. 4(a) in he main article.

**File Name:** **Supplementary Movie 2**

**Description:** Visualization of the numerical simulation showing the propagation of the magnetic and velocity pulses in the flux tube. Vertical lines are magnetic field lines of the flux tube, with colours denoting the local horizontal magnetic field perturbation δBa. The black-red rendering shows the local rotating speed vr within a vertical slice at x=0 km. The bottom layer shows part of the SOT FG-blue photospheric intensity observations from 06:01:35 UT to 06:03:36 during which a swirl with a lifetime of ~109 s was detected at the centre of the layer in the intergranular lane.

**File Name: Supplementary Movie 3**

**Description:** Visualization of the toy model constructed in the Supplementary Discussion. The grey cylinder shows the surface of the constructed flux tube with colored lines as its magnetic field lines. Colours along the magnetic field lines denote azimuthal magnetic field strengths. Different colours in the central horizontal disk represent different local plasma densities.
